# Editorial: Insights in Structural Biology: 2021

**DOI:** 10.3389/fmolb.2022.1020473

**Published:** 2022-10-07

**Authors:** Caterina Alfano, Annalisa Pastore, Piero Andrea Temussi

**Affiliations:** ^1^ Structural Biology and Biophysics Unit, Fondazione Ri.MED, Palermo, Italy; ^2^ European Synchrotron Radiation Facility, Grenoble, France; ^3^ University of Naples “Federico II”, Napoli, Italy

**Keywords:** structural biology, molecular dynamics, NMR, mass spectrometry, *in vivo*

The common theme of this Research Topic (RT) was to discuss examples of the most recent achievements made by scientists in the fast-growing field of Structural Biology. In particular, the goal of this special edition Research Topic was to shed light on the progress made in the past decade in Structural Biology and to provide an overview of the state-of-the-art of the field. We think that the articles received will inspire, inform and provide new directions and guidance to researchers.

One of the fields in which Structural Biology has become mature in within the last few years covers new approaches in computational methods.

Two of the papers accepted in the present research topic are those by the group of Patrick Senet. Both papers use a new software that was developed in this laboratory to extract conformational properties of proteins on the basis of the coordinates of α-carbons. The program is called CUTABI (CUrvature and Torsion based Alpha-helix and Beta-sheet Identification) and identifies residues in α-helices and β-sheets using solely α-carbon coordinates. This algorithm is ideal for the difficult cases in which the content of secondary structure is not directly determined.

The first of these papers (RT_1) by Guzzo et al. (2021) deals with α-synuclein and three single point mutations (A39P, A53T and E46K) of this protein related to familial forms of Parkinson disease (PD). The authors addressed the problem of identifying differences in conformational tendencies, possibly related to different tendencies to aggregate in β-sheet structures, that is the hallmark of PD. They calculated two-dimensional probability density maps and evaluated their differences by molecular dynamics trajectories. In the observed conformational states, the authors found some with a two-phase characteristics and a homogeneous (B, only β-sheets) and a heterogenous phase (HB, a mixture of α-helices and β-sheets). The B state is populated by 40% in the wild-type and in two mutants (A35T and A30P) whereas it is present only as a 25% population in the A53T mutant. The A53T mutant has also a rather larger propensity to forming helices than the wild-type and other mutants. The authors concluded that the equilibrium between the different conformations of the α-synuclein monomer is modified by the missense mutations in a subtle way. It is highly significant that these authors could reach this conclusion in the case of an intrinsically unfolded protein because other parameters, such as the average gyration radius, could not be used to discriminate among conformational ensembles. The introduction of the algorithm CUTABI is promising for studies of intrinsically unfolded proteins.

The second paper by the same group (RT_9) focused on the properties of dimers of α-synuclein and the three single point mutations discussed in RT_1, i.e., A30P, A53T and E46K (Guzzo et al.). The authors found that there are two main segments in the sequence, with higher tendency to aggregate in the early stages of dimerization. The main result is that dimers of α-synuclein and of the three single point mutations do not adopt the same fold motif in fibrils but form disordered aggregates and a minority of prefibrillar dimers with intra- and inter-molecular β-sheets. This second contribution reinforces the importance of the development of CUTABI, an algorithm that could be useful in the investigation of unfolded proteins.

Most of the other papers of this RT are based on traditional spectroscopic methods. The paper presented by the Boeckmann’s group (RT_2, Fogeron et al.) used mainly solution nuclear magnetic resonance (NMR) but also circular dichroism spectroscopy and mass spectrometry to investigate the importance of phosphorylation sites in the large envelope protein of human hepatitis virus (HBV). The large envelope protein occupies a central role for interactions with the HBV cellular receptor but also with the HBV capsid, the Hsc70 chaperone and cellular membranes during fusion. Using mass spectrometry and NMR, the authors established the phosphorylation patterns of human HBV L protein. Contrary to what was known for the analogous avian virus, the locations identified for the human virus might play a functional role because they were found at strategic sites previously predicted to be crucial for interactions of the large protein. Altogether, the paper by Fogeron et al. shows the impact of spectroscopic methods in the study of even large proteins.

The paper presented by Ami et al. (RT_3) is a review of the use of infrared (IR) spectroscopy to the study of aggregation phenomena in complex biological systems, such as intact cells and tissues (Ami et al.). IR spectroscopy has been regarded for many years as a physicochemical technique mainly useful for small molecules, but recently the use of this spectroscopy has been extended to *in situ* characterization of the conformational properties of protein aggregates and to the investigation of other biomolecules surrounding aggregates, e.g., those surrounding amyloids. The characterization of protein aggregates in their natural environment may help to narrow the gap between the knowledge of amyloid aggregation mechanisms *in vitro* and *in vivo*.

NMR spectroscopy is also the main technique used in the paper (RT_7) by Spyroulias and coworkers (Birkou et al.). These authors studied the impact of single nucleotide polymorphisms on Arkadia (RNF111), an E3 ubiquitin ligase that enhances transforming growth factor-beta (TGF-β) signalling by targeting negative regulators for degradation. In particular, a single nucleotide polymorphism generated on the enzymatic ring of Arkadia the substitution of Arginine 957 to cysteine. Detailed NMR investigations showed that the R957C mutation changes the electrostatic properties of the RING domain of Arkadia, without significant effects on the structure of the region. However, the R957C Arkadia mutant exhibits increased enzymatic activity, in agreement with increased aggressive and metastatic behaviour of Arkadia within tumor cells.

Several spectroscopic and computational methods are quoted in the review (RT_4) by Fedeles et al. that describes the relevance of tautomeric dynamics in nucleic acids and in antiviral nucleoside analogs. The chemical versatility of nucleic acids is due in part to the phenomenon of nucleobase tautomerism, because the bases can adopt multiple isomeric forms, known as tautomers. Tautomers of nucleobases refer to structural isomers that differ from one another by the position of protons. Thus, by altering the position of protons, tautomerism has profound effects on the biochemical processes involving nucleic acids. For instance, the transient formation of minor tautomers during replication could generate spontaneous mutations. In the review, the authors discuss the consequences of tautomerism on the fidelity of DNA replication but also on RNA systems such as riboswitches and self-cleaving ribozymes.

NMR and other physico-chemical techniques are at the basis of a paper (RT_8) by the Polshakov’s group (Mariasina et al.). These authors tackled a complex problem related to a genetic disorder (Williams-Beuren syndrome) associated with the hemizygous deletion of several genes in chromosome 7. Malfunction of 26 proteins inducing multisystemic failure is well established for most of them, but remains elusive for methyltransferase WBSCR27. Considering the complexity of the problem, the authors tried several approaches. They first tried to find a substrate of methylation catalyzed by WBSCR27 by constructing mouse cell lines with a WBSCR27 gene knockout and studied these cells by several molecular biology and mass spectrometry techniques. In all cases, neither a direct substrate was identified nor the protein partner was detected. To reveal the nature of the putative methylation substrate, the authors determined the solution structure of WBSCR27 in the apo form and in a complex with Sadenosyl-l-homocysteine. The protein core adopts a canonical Rossman fold with a disordered N-terminus. Binding to S-adenosyl-l-homocysteine induces a transition to a well-formed binding state. The structure of the binding site suggests potential substrates of WBSCR27 methylation to be probed in further studies.

The mini review (RT_6) by Garnett and Atherton, while still quoting the importance of recent improvements in solid state NMR, is centred on the spectacular advances in cryogenic electron microscopy (cryo-EM) (Garnett and Atherton). The review deals with the notoriously difficult study of proteins that form highly polymeric and filamentous assemblies using high resolution structural techniques. The study of eukaryotic microtubules and bacterial pili are good examples, and the review gives an overview of the advances that have been made for these two systems.

Last but not least, the review (RT_5) by Bellotti’s collaborators tackles a general problem of structural studies on aggregation (Faravelli et al.). Their review focuses on the need to narrow the gap between *in vitro* and *in vivo* mechanisms when studying amyloid formation by globular proteins. Although all systemic amyloidoses are characterized by amyloid deposition, they are clinically heterogeneous because they affect different organs. It is thus essential to elucidate the structural events determining the protein metamorphosis from their globular to fibrillar state. Many studies have shown how to transform proteins into fibrillar polymers *in vitro* but the events occurring *in vivo* are more complex. Reviewing the major scientific attempts to understand the amyloidogenic metamorphosis of globular proteins in systems of increasing complexity may help to bridge the gap between the experimental models and the actual pathogenic events.

Overall, the nine papers of this exciting RT provide an up-to-date view of the most recent achievements made by scientists in the evergrowing field of Structural Biology.

